# Indirect genetic effects on the relationships between production and feeding behaviour traits in growing Duroc pigs

**DOI:** 10.1017/S1751731119002179

**Published:** 2019-10-01

**Authors:** W. Herrera-Cáceres, M. Ragab, J. P. Sánchez

**Affiliations:** 1 Genetica i Milloa Animal, Institut de Recerca i Tecnologia Agroalimentàries (IRTA), Torre Marimon s/n, Caldes de Montbui, Barcelona 08140, Spain; 2 Poultry Production Department, Kafr El Sheikh University, Kafr El Sheikh 33516, Egypt

**Keywords:** feed efficiency, genetic parameters, genetic selection, individual intake system and social interactions

## Abstract

Performance and feeding behaviour traits in growing pigs could be affected by social interaction effects when animals are raised in group. So, properly knowing the genetic correlations between direct and social interaction effects among performance and feeding behaviour traits could improve the accuracy of the genetic evaluations. Our aim was to explore the role of feeding behaviour traits (**FBT**) and indirect genetic effects (**IGEs**) in the genetic evaluations of growing pigs. Thus, genetic parameters were estimated for production traits (**PT**): average daily gain, average daily feed consumption, feed conversion ratio and backfat thickness; as well as for FBT: average daily feeding rate, average daily feeding frequency, average daily occupation time and average daily time between consecutive visits. Traits were recorded in 1144 Duroc pigs during the fattening period. Two bivariate models were fitted: classic animal model and an animal model fitting IGE. Estimations were done following Bayesian procedures. Heritability estimates obtained with classic animal model for all studied traits were medium-high. The additional heritable variation captured by IGE supposed that the ratios of total genetic variance to phenotypic variance (*T*^*2*^) were higher than the heritability estimates obtained with the classic model, except for occupation time trait, when a lower value (0.20 ± 0.19) was estimated. This is due to a high and negative correlation between IGE and direct genetic effects (**DGEs**) of this particular trait (−0.78 ± 0.27). Results from classic animal model do not evidence a clear role of FBT to improve the accuracy of breeding value predictions for PT; only average daily feeding rate seems to show a positive correlation (around 0.50 to 0.60) with average daily gain, average daily feed consumption and backfat thickness. However, when IGE model was fitted, the number of estimates of genetic correlations between FBT and PT showing a relevant magnitude increased, generally for the correlations between IGE of FBT and DGE of PT; or particularly for the correlations between IGE of average daily feeding frequency, and the IGE of all the PT, except average daily gain. Thus, in evaluations using the animal model with IGE fitted, the inclusion of FBT could aid the improvement of the accuracy of breeding value predictions for PT. This is a consequence of the improved genetic relationships between traits that can be fitted when considering such models.

## Implications

The accuracy of breeding value prediction for production traits could be improved if correlated traits are jointly considered in evaluations, and such traits could be feeding behaviour traits. Feeding behaviour traits as well as production traits are recorded in group housed animals; thus animal models fitting indirect genetic effects could be a tool to account for this fact. Our results show that these models are preferable over the traditional animal model for both production traits and feeding behaviour traits, and particularly when the evaluation is done using animal models fitting indirect genetic effects. The breeding value prediction accuracy for production traits could be increased in such a joint evaluation with feeding behaviour traits, since certain correlations between genetic effects across traits show relevant magnitudes. In addition, these animal models fitting indirect genetic effects offer an improved correlation structure and alternative selection indexes.

## Introduction

The relationship between production and behaviour traits is particularly relevant when the animals are reared sharing a common space, as it commonly occurs in swine (Chen *et al*., 2007; Ellen *et al*., 2014). Under this housing system, although it could be difficult or costly, obtaining measures of individualised consumption is possible at certain levels of a breeding schema, that is, selection nucleus. This can be done thanks to the availability in the market of feeding devices allowing individual feed intake recording (Eissen *et al*., 1998). These devices also allow for the recording of individual feeding behaviour traits (**FBT**; Young, 2012), which might play an important role in any selection programme to improve growth or feed efficiency in pigs as far as they show relevant heritability and moderate genetic correlation with traits of direct economic importance (Labroue *et al*., 1997; Young *et al*., 2011). However, these FBT, as they would be recorded in group-housed animals, could be subject to the same issues of interactions between group mates as happen with performance traits (Griffing, 1967; Bijma and Wade, 2008). In this context, animal models fitting indirect genetic effects (**AM-IGEs**) could be relevant in the exploration of both direct and indirect correlations between PT and FBT. These models assume that the phenotype of an individual is influenced by both genetic effects in the individual itself (direct genetic effect (**DGE**)) and genetic effects in its pen mates; these are known as indirect genetic effects (**IGEs**; Bijma and Wade, 2008). The objective of this work was to estimate genetic parameters of PT and FBT during the fattening period in a Duroc line considering either traditional animal models or AM-IGE, with the aim of assessing the role that these FBT might play in a breeding programme for feed efficiency.

## Material and methods

### Animal and management

Performances during growing-fattening period (10 to 26 weeks of age) on 1144 Duroc pigs were recorded in 10 batches from 2007 to 2017 at the Center of Porcine Evaluation (Monells, Girona, Spain) using IVOG feeding stations (Insentec, Markenesse, the Netherlands) with a single-space feeder for each pen. Animals represented 430 litters sired by 85 boars and from 399 different sows. The completely known pedigree comprises 5077 individuals from 671 sires and 3264 dams. This Duroc line was founded in 1991 (Tibau *et al*., 1999) and selected as multi-purpose line until the mid-2000s, when the selection index applied changed with the aim of specialising the population as a maternal line, since then most of the weight in the index has been assigned to prolificacy and backfat thickness (**BF**). For our study, the animals with phenotypic information were housed in 97 different pens having on average (SD) 12.0 (1.5) animals per pen, the size of the pens ranged from 7 to 15 animals. The age at the start of the control was 70 (6) days old and the age at the end of the control was 177 (9) days old. In all the batches animals were fed *ad libitum* on a standard diet satisfying their requirements. As our data cover a long period of time (10 years), certain variation in the diet composition exists across batches. Nevertheless, the average nutrient composition across batches and fattening phases (growing (20 to 60 kg) and finishing (60 to 100/110 kg)) was the following: 15.0 % CP, 4.7 % fibre, 4.5 % fat, 0.9 % lysine and 0.3 % methionine, having 3200 kcal of metabolic energy per kilogram of feed. Variability in the diet across batches will be statistically accounted for by fitting a batch effect in the models. A number of FBT as well as daily feed intake and other production traits (**PT**) were recorded.

### Production trait description

Individual live BW (kg) and BF (mm) were recorded during the fattening period; the number of measures performed for both traits on each animal ranged from 4 to 11. Backfat thickness was measured at 5 cm of the midline between the third and fourth last ribs using ultrasounds (PIGLOG 105, SFK-Technology, Herlev, Denmark). A linear regression model within each animal dataset was fitted individually to adjust BF to 180 days of age (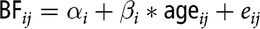). We used this age in spite of the fact that the average age at the end of the fattening period is a few days less (177) because 180 days is the reference age used for the selection of the line, the difference with most of the individual ages at the end of the fattening period is small (no more than 15 days) and around these ages a fairly linear pattern was observed. Average daily gain (**ADG**, kg/day) was also individually computed using a within-animal linear regression model of live BW on age, that is, 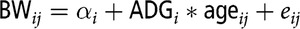. Average daily feed consumption (**ADC**, kg/day) was computed following Sánchez *et al*. (2017); missing daily feed intake records were predicted using animal-nested random polynomial regressions, and the ADC for a particular animal was defined as the mean of all the available individual daily feed intake records. Feed conversion ratio (**FCR**) was computed as ADC divided by ADG.

### Feeding behaviour trait description

The electronic feeders directly report only the duration of each visit to the feeder, from this information, and from the daily occupation pattern, four FBT referring to the completely fattening period were derived. In an initial edition step, hourly blocks were defined, and for each hour and individual, mean feeding rate (g/min), total feed intake (g), number of visits to the trough (number of visits) and total time occupying the trough (s) were calculated. From these hourly basis measurements, daily aggregates for feeding rate, number of visits and time occupying the trough were obtained; for the case of feeding rate the aggregation was only done averaging hours in which feed intake information was recorded; and for the case of number of visits and occupation time the aggregation was calculated by summing. In addition, average daily time between consecutive visits to the feeder was computed from hourly records; this was done first by computing the hourly intervals between day hours having visits to the feeder and then averaging within animal and day information. Finally, fattening period measurements were obtained as within-animal average across daily aggregates. The resulting traits to be analysed were average daily feeding rate (**FR**, g/min), average daily feeding frequency (**FF**, visits), average daily occupation time (**OT**, min) and average daily time between consecutive visits (**FInt**, h). Table 1 presents basic statistics of all the aforementioned traits.

**Table 1:** Descriptive statistics for performance and feeding behaviour traits of growing Duroc pigs

Category	Trait	No. of observations	Minimum	Mean	Maximum	SD
Behaviour	FR, g/min	1144	15.28	38.60	65.14	7.41
	OT, min	1144	33.02	60.72	103.49	10.27
	FF, visits	1144	3.54	10.11	24.88	2.98
	FInt, h	1144	1.64	3.93	9.89	1.03
Performance	ADG, kg	1144	0.22	0.82	1.07	0.09
	ADC, kg	1144	0.86	2.31	3.67	0.37
	FCR	1144	2.07	2.77	3.89	0.24
	BF, mm	1144	6.44	18.19	32.74	4.40

FR = average daily feeding rate; OT = average daily occupation time; FF = average daily feeding frequency; FInt = average daily time between consecutive visits; ADG = average daily gain; ADC = average daily consumption; FCR = feed conversion ratio; BF = backfat thickness.

### Statistical models

Two bivariate animal models either considering or not IGEs were implemented. For all studied traits, the same systematic effects were fitted: batch (10 levels), age at the end of the control period (covariate) and number of pigs in the pen (covariate). In addition, pen and litter effects were also included, as well as the additive genetic effect. All the previous effects were fitted in the classic animal model (**AM**), whereas in the AM-IGE, the genetic component was divided into DGEs and IGEs (Bijma and Wade, 2008; Duijvesteijn *et al*., 2012). This last case is the most complex model studied and is represented by the following equation:
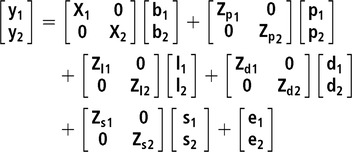
where ***y_1_*** and ***y_2_*** are the vector of observations for the first and second traits, ***b*** is a vector of systematic effects with incidence matrix ***X***; ***p*** is a vector of pen effects with incidence matrix ***Z_p_***; ***l*** is a vector of litter effects with incidence matrix ***Z_l_***; ***d*** is a vector of DGEs with incidence matrix ***Z_d_***; ***s*** is a vector of IGEs with incidence matrix ***Z_s_***. The elements of ***Z_s_*** are 1 for the records from animals sharing the same pen and 0 otherwise; ***e*** is a vector of residuals.

Under AM-IGE model, each individual interacts with *n–1* of its group members where *n* is the group size. Under this model, the total breeding value of an individual *i* is equal to 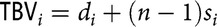 The total breeding value variation among individuals is 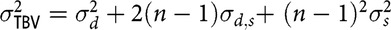. The ratio between the total breeding value variation and the total phenotypic variance (*T* ^2^) might exceed one; because the total phenotypic variation is 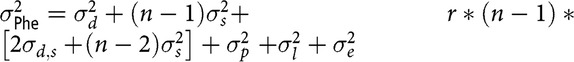, where 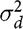 is the DGE variance, 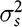 is the IGE variance, 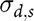 is the covariance between DGEs and IGEs, 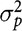 is the pen effect variance, 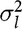 is the litter effect variance, 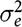 is the error variance and *r* is the mean kinship coefficient between pen members; in our case this coefficient was equal to 0.13. Bijma *et al*. (2007) already presented all these parameter definitions.

Bayesian procedures were used to estimate model parameters, the prior pdistribution of pen, litter effect and the residuals were: 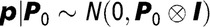 ; 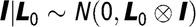 and 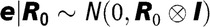, respectively, where ***I*** represents identity matrices of appropriate dimensions, and ***P***_0_, ***L***_0_ and ***R***_0_ are covariance matrices of dimension 2, containing pen, litter and residual (co)variances of the two traits studied at the time and ⊗ denotes the kronecker product. All factors were assumed to be independent, except DGEs and IGEs. For them the assumed prior distribution was: 
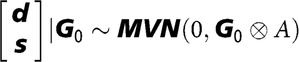
, where ***A*** is the numerator relationship matrix between individuals, ***G***_0_ is the covariance matrix, containing the following elements:
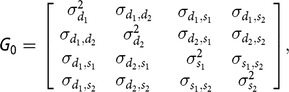
where 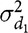 is the direct genetic variance of the first trait, 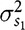 is the indirect genetic variance for the second trait, 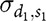 and 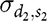 are the covariance between DGE and IGE, for the first and the second traits, respectively, 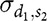 is the covariance between DGE of the first trait and IGE of the second trait, 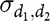 is the covariance between DGE of the two traits and finally 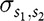 is the covariance between IGE of the two traits. In the Bayesian analysis performed, flat priors were assumed for systematic effects (***b***) and for all the variance components previously described: ***G***_0_, ***P***_0_, ***L***_0_ and ***R***_0_.

The marginal posterior distributions of all unknown parameters were obtained using the Gibbs Sampling algorithm, with the programme gibbs2f90 (Misztal *et al*., 2002). Chains of 1 000 000 samples were run, and the first 100 000 iterations were discarded in order to allow the algorithm to reach convergence to the marginal posterior distributions. Then, one sample every 10 iterations was saved; thus the autocorrelation of the chain was reduced. Convergence of the Markov chains was assessed by visual inspection of the trace plots. Finally, deviance information criterion (**DIC**, Spiegelhalter *et al*., 2002) was used in order to compare the models based on their fitting quality, but penalising by model complexity.

## Results

### Heritabilities, ratios of litter and pen variances to phenotypic variance and magnitude of indirect genetic effects

The first remarkable result is that for all considered traits, IGE plays an important role. This is evidenced by the fact that DIC values are always substantially lower (<18) in the AM-IGE than in the AM (Table 2). In addition to this, the magnitude of *T* ^*2*^ was higher than the heritability from the AM for all the studied traits, except for the occupation time in the feeder (posterior mean ± posterior SD) (0.20 ± 0.19). The low value of *T* ^*2*^ for the occupation time is related to the fact that a high and negative genetic correlation was estimated between IGE and DGE for this particular trait (−0.78 ± 0.27) with a probability greater than 95 % of being lower than zero. This correlation means that individuals with a positive DGE, for animals staying longer in the feeder, tend to have negative IGE, forcing their pen mates to stay for less time. Litter effect variance represented 7 % to 14 % phenotypic variance for PT and 2 % to 6 % phenotypic variance for FBT. The ratio of pen variances to phenotypic variances ranged from 4 % to 13 % for PT and from 6 % to 13 % for FBT.

**Table 2:** Marginal posterior means (SD) of ratio of variances and DIC of growing Duroc pigs using univariate animal models with and without indirect genetic effects

	AM		AM-IGE						
Trait	*h^2^*	DIC	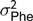	*T^2^*	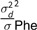	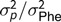	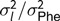	*r*(S,D)	DIC
FR	0.30 (0.08)	7304.83	48.73 (3.38)	0.39 (0.29)	0.32 (0.08)	0.13 (0.04)	0.04 (0.03)	−0.19 (0.70)	7282.56
OT	0.23 (0.10)	8097.19	84.98 (4.82)	0.20 (0.19)	0.27 (0.10)	0.08 (0.03)	0.06 (0.04)	−0.78 (0.27)*	8055.65
FF	0.48 (0.09)	4871.82	8.72 (0.83)	0.93 (0.49)	0.46 (0.09)	0.12 (0.06)	0.03 (0.03)	−0.21 (0.41)	4803.53
FInt	0.47 (0.08)	2835.14	1.09 (0.07)	0.67 (0.30)	0.47 (0.09)	0.06 (0.03)	0.02 (0.02)	−0.21 (0.47)	2792.88
ADG	0.19 (0.08)	2562.15	0.68 (0.04)	0.22 (0.18)	0.22 (0.09)	0.04 (0.02)	0.14 (0.04)	−0.58 (0.42)	2541.09
ADC	0.22 (0.08)	5246.81	7.62 (0.52)	0.29 (0.29)	0.25 (0.08)	0.11 (0.04)	0.08 (0.04)	−0.57 (0.47)	5219.54
FCR	0.21 (0.09)	4418.22	4.00 (0.35)	0.55 (0.43)	0.24 (0.09)	0.13 (0.05)	0.07 (0.04)	−0.10 (0.70)	4400.04
BF	0.32 (0.10)	5579.10	10.75 (0.72)	0.51 (0.28)	0.35 (0.11)	0.05 (0.03)	0.09 (0.04)	−0.02 (0.61)	5553.75

AM = classic animal model; AM-IGE = Animal model including indirect genetic effects; FR = average daily feeding rate; OT = average daily occupation time; FF = average daily feeding frequency; FInt = average daily time between consecutive visits; ADG = average daily gain; ADC = average daily consumption; FCR = feed conversion ratio; BF = backfat thickness. *h^2^* = heritability; DIC = deviance information criterion; 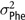 = total phenotypic variance; 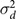 = direct genetic effect variance; 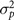 = pen variance; 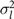 = litter variance; *T^2^* = the ratio between the total breeding value variation and 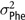; 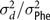 = portion of phenotypic variance explained by direct genetic effect; 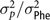 = portion of phenotypic variance explained by pen effect; 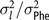 = portion of phenotypic variance explained by litter effect; *r*(S,D) = genetic correlation between direct and indirect genetic effects of the same trait.

* Probability of being greater than 0 > 0.95 or <0.05.

### Estimated phenotypic and genetic correlations using classic animal model

Although the relevance of IGE is clear, this section presents the genetic and phenotypic correlations between traits obtained with the traditional AM (Table 3). These correlations are presented to describe a putative social structure in the pen, and also to establish a basis for later discussion on the genetic correlations between traits based on the AM-IGE. With regard to the phenotypic correlations nearly all of them can be said to be statistically different from zero. The feeding rate and the occupation time are positively correlated at phenotypic level with all PT, ranging from 0.11 to 0.42. These correlations between the feeding frequency and all the PT are much lower and negative, as high as −0.11. Within FBT, the feeding rate was positively correlated with the time between visits (0.10) and negatively correlated with occupation time (−0.65) and feeding frequency (−0.11). The phenotypic correlation between occupation time and feeding frequency was estimated to be positive (0.20), while negative with the time between visits (−0.28). Finally the estimated phenotypic correlation between feeding frequency and time between visits was −0.60. Regarding the genetic correlations, ADC was positively correlated with the other PT: FCR (0.66 ± 0.22), BF (0.64 ± 0.16) and ADG (0.80 ± 0.13). The genetic correlation between BF and ADG was positive (0.51 ± 0.25). Finally, the genetic correlations between FCR and both ADG and BF had large estimation errors; thus, it was not possible to clearly define whether their values are positive or negative, but it seems to be of lower magnitude than the rest of the genetic correlations. Only two genetic correlations out of the six estimated between FBT showed a magnitude statistically different from zero (above diagonal in Table 3). This was the case between feeding rate and occupation time (−0.76 ± 0.16), and between feeding frequency and the time between visits (−0.78 ± 0.09). The other genetic correlations between FBT were low and with undefined signs due to their high estimation errors. The role that FBT might play on the prediction of breeding value for PT was defined by exploring the genetic correlation estimates between PT and FBT (Table 3). Only feeding rate was positively correlated at genetic level with most of the PT (ADG, ADC and BF).

**Table 3:** Genetic (above diagonal) and phenotypic (below diagonal) correlations estimated of growing Duroc pigs using the classic animal model

Trait	FBT				PT			
	FR	OT	FF	FInt	ADG	ADC	FCR	BF
FR		−0.76 (0.16)*	0.22 (0.20)	−0.31 (0.20)	0.60 (0.24)*	0.64 (0.22)*	0.40 (0.29)	0.53 (0.24)*
OT	−0.65 (0.02)*		0.27 (0.25)	−0.33 (0.26)	−0.24 (0.38)	−0.30 (0.37)	−0.28 (0.39)	−0.33 (0.36)
FF	−0.11 (0.04)*	0.20 (0.04)*		−0.78 (0.09)*	0.11 (0.26)	0.16 (0.24)	0.15 (0.25)	0.17 (0.22)
FInt	0.10 (0.04)*	−0.28 (0.03)*	−0.60 (0.03)*		−0.11 (0.28)	−0.09 (0.25)	0.04 (0.28)	−0.32 (0.23)
ADG	0.38 (0.03)*	0.16 (0.04)*	−0.11 (0.04)*	0.10 (0.04)*		0.80 (0.13)*	0.14 (0.41)	0.51 (0.25)*
ADC	0.42 (0.03)*	0.20 (0.04)*	−0.11 (0.04)*	0.04 (0.04)	0.82 (0.01)*		0.66 (0.22)*	0.64 (0.16)*
FCR	0.16 (0.04)*	0.11 (0.04)*	−0.03 (0.04)	−0.05 (0.04)	−0.05 (0.04)	0.52 (0.03)*		0.41 (0.33)
BF	0.29 (0.03)*	0.16 (0.04)*	−0.09 (0.04)*	−0.04 (0.04)	0.59 (0.03)*	0.68 (0.02)*	0.31 (0.03)*	

FBT = feeding behaviour traits; PT = production traits; FR = average daily feeding rate; OT = average daily occupation time; FF = average daily feeding frequency; FInt = average daily time between consecutive visits; ADG = average daily gain; ADC = average daily consumption; FCR = feed conversion ratio; BF = backfat thickness.

* Probability of being greater than 0 > 0.95 or <0.05.

### Estimated genetic correlations using animal model fitting indirect genetic effects

Estimated genetic correlations between DGE for PT (above diagonal in Table 4) followed a similar structure to that of the genetic correlations between additive genetic effects obtained with the AM. The only remarkable difference was observed between FCR and BF; in this case the genetic correlation can be declared as positive (0.45 ± 0.19). The estimated genetic correlations between DGE for FBT (above diagonal in Table 4) were similar to the correlation structure between genetic effects from the AM (Table 3). With regard to the estimated genetic correlations between IGE of PT (below diagonal in Table 4), a similar correlations structure, with respect to their directions and also partially to their magnitudes, as that for DGE was observed. However, the estimated genetic correlations between IGE of FBT showed a different pattern (below diagonal in Table 4). The estimate of IGE correlation between the time between visits and occupation time was clearly negative (−0.74 ± 0.19) and the genetic correlation between IGE of feeding rate and the time between visits was also negative (−0.51 ± 0.25). The DGE correlation estimates between these two pairs of traits were not statistically different from zero. Regarding the estimated genetic correlations between DGE for PT and FBT (above diagonal Table 4), the structure was almost the same as that indicated for breeding values from AM; that is, the only FBT related with PT was feeding rate. The image with respect to the relationships between PT and FBT regarding IGE (below diagonal in Table 4) was different; in this case, IGE for feeding frequency was negatively correlated with IGE of all PT, except ADC. Therefore, animals that tend to force their mates into going to the feeder very frequently also produce an effect on their mates that reduces their ADG, BF and FCR. Similarly, animals that tend to increase the time between consecutive visits to the feeder of their mates also produce a reduction in their mates’ BF, and the genetic correlation between IGE of the time between visits and BF is close to −1. In addition, the relationships among IGEs show that animals tending to reduce the occupation time of their mates also reduce their ADG, and the estimated genetic correlation between IGE of the occupation time and ADG was 0.59 ± 0.25. Finally, it seems that animals forcing their mates to eat at a higher pace also produce an increase in their mates’ FCR. This is because a positive correlation was estimated between IGEs of feeding rate and FCR (0.61 ± 0.23).

**Table 4:** Genetic correlations estimated among direct genetic effects (above diagonal) and among indirect genetic effects (below diagonal) for production and feeding behaviour traits of growing Duroc pigs using animal model fitting indirect genetic effects

Trait	FBT				PT			
	FR	OT	FF	FInt	ADG	ADC	FCR	BF
FR		−0.71 (0.11)*	0.25 (0.17)	−0.22 (0.17)	0.45 (0.19)*	0.47 (0.17)*	0.24 (0.21)	0.45 (0.18)*
OT	−0.90 (0.14)*		0.31 (0.19)	−0.25 (0.18)	−0.01 (0.24)	0.02 (0.24)	0.06 (0.25)	−0.03 (0.23)
FF	−0.24 (0.29)	0.12 (0.32)		−0.72 (0.09)*	0.06 (0.21)	0.06 (0.19)	0.11 (0.19)	0.11 (0.18)
FInt	−0.51 (0.25)*	−0.74 (0.19)*	−0.99 (0.01)*		−0.09 (0.18)	−0.14 (0.17)	0.20 (0.19)	−0.21 (0.18)
ADG	−0.10 (0.35)	0.59 (0.25)*	−0.82 (0.19)*	−0.29 (0.30)		0.83 (0.08)*	0.13 (0.25)	0.55 (0.18)*
ADC	−0.14 (0.37)	0.10 (0.35)	−0.42 (0.25)	−0.39 (0.29)	0.70 (0.21)*		0.61 (0.16)*	0.73 (0.11)*
FCR	0.61 (0.23)*	−0.35 (0.29)	−0.74 (0.14)*	0.13 (0.28)	0.06 (0.38)	0.64 (0.23)*		0.45 (0.19)*
BF	0.16 (0.37)	0.28 (0.34)	−0.89 (0.12)*	−0.97 (0.03)*	0.67 (0.26)*	0.55 (0.26)*	0.75 (0.25)*	

FBT = feeding behaviour traits; PT = production traits; FR = average daily feeding rate; OT = average daily occupation time; FF = average daily feeding frequency; FInt = average daily time between consecutive visits; ADG = average daily gain; ADC = average daily consumption; FCR = feed conversion ratio; BF = backfat thickness.

* Probability of being greater than 0 > 0.95 or <0.05.

In order to have a full picture of the genetic structure between traits when considering AM-IGE for genetic evaluation purposes, it is necessary to study the genetic correlations between traits combining IGE and DGE (Table 5). Among PT the only correlation that was statistically different from zero involved DGE of FCR and IGE of ADG (0.73 ± 0.14). This positive correlation means that animals with a favourable genetic value to induce higher growth rates in their mates also tend to have genetic effects that worsen its own FCR. With regard to FBT, the only correlations between IGE and DGE seeming to be statistically different from zero were that involving DGE of the time between visits and IGE of feeding rate (−0.54 ± 0.20), and those between DGE of feeding frequency and IGE of feeding rate and occupation time (0.68 ± 0.11 and 0.37 ± 0.17, respectively); that is, animals with breeding values that increase their own feeding frequency also have genetic effects that increase feeding rate and occupation time of their mates. With respect to the correlations involving IGE of PT and DGE of FBT, all were relatively weak, and the observed estimation errors do not allow for properly declaring whether they are positive or negative. However, regarding the correlations between IGE of FBT and DGE of PT, many of them were statistically different from zero. For example, DGE of FCR correlates with IGE of all FBT except for feeding rate; as in some cases this correlation was positive (with occupation time and the time between visits), but in another case it was negative (with feeding frequency). Yet another case in which important correlations were detected involved DGE of ADC and IGE of feeding rate, feeding frequency and the time between visits. Regarding DGE of BF, it only seemed to be positively correlated with IGE of feeding rate. Finally, it has to be remarked that DGE of ADG was positively correlated with IGE of the time between visits (0.88 ± 0.10) and IGE of feeding rate (0.89 ± 0.11); animals with favourable genetic effects for their own growth tend to carry genetic effects that incline their mates to have longer interval between consecutive visits and to show a high eating speed.

**Table 5:** Genetic correlations estimated between IGE and DGE for different combinations between production and feeding behaviour traits of growing Duroc pigs using animal model fitting indirect genetic effects

Trait	FBT				PT			
	FR_DGE_	OT_DGE_	FF_DGE_	FInt_DGE_	ADG_DGE_	ADC_DGE_	FCR_DGE_	BF_DGE_
FR_IGE_		0.41 (0.32)	0.68 (0.11)*	−0.54 (0.20)*	0.89 (0.11)*	−0.82 (0.15)*	−0.15 (0.34)	0.47 (0.18)*
OT_IGE_	0.28 (0.31)		0.37 (0.17)*	0.33 (0.23)	−0.19 (0.27)	−0.15 (0.23)	0.43 (0.21)*	0.01 (0.25)
FF_IGE_	−0.15 (0.30)	−0.10 (0.31)		0.16 (0.25)	−0.09 (0.36)	−0.53 (0.24)*	−0.55 (0.23)*	−0.26 (0.32)
FInt_IGE_	0.08 (0.32)	0.13 (0.34)	0.28 (0.24)		0.88 (0.10)*	0.98 (0.02)*	0.57 (0.19)*	0.06 (0.28)
ADG_IGE_	0.05 (0.35)	−0.11 (0.34)	−0.06 (0.32)	−0.30 (0.26)		−0.07 (0.26)	0.73 (0.14)*	0.29 (0.27)
ADC_IGE_	0.11 (0.34)	−0.05 (0.36)	0.15 (0.27)	−0.26 (0.28)	−0.42 (0.31)		0.24 (0.25)	0.07 (0.22)
FCR_IGE_	0.13 (0.36)	−0.04 (0.33)	0.16 (0.26)	−0.003 (0.26)	−0.27 (0.35)	−0.33 (0.32)		−0.3 (0.34)
BF_IGE_	−0.27 (0.32)	−0.03 (0.36)	−0.14 (0.32)	0.03 (0.27)	−0.17 (0.37)	−0.29 (0.32)	0.06 (0.43)	

IGE = indirect genetic effect; DGE = direct genetic effect; FBT = feeding behaviour traits; PT = production traits; FR = average daily feeding rate; OT = average daily occupation time; FF = average daily feeding frequency; FInt = average daily time between consecutive visits; ADG = average daily gain; ADC = average daily consumption; FCR = feed conversion ratio; BF = backfat thickness.

* Probability of being greater than 0 > 0.95 or <0.05.

## Discussion

Our results clearly address the important role of IGE on the definition of traits recorded in animals raised in groups. This finding was already reported for PT in other pig populations (Bergsma *et al*., 2008; Chen *et al*., 2008; Nielsen *et al*., 2018). Until now, at least to our knowledge, there were no reports on the relevance of the IGE for FBT. These traits have been always considered using AM, and their estimated heritabilities were moderate to high (Young 2012; Do *et al*., 2013; Lu *et al*., 2017). When we applied the AM to these traits, we did also find, in general, high heritabilities, ranging from 0.23 to 0.47. Although our model choice criteria favoured the model AM-IGE, the estimation errors of certain parameters, in particular the correlation between IGE and DGE, were high, and only for the case of trough occupation time its negative value can be statistically defined. This indicates that animals with genetic effects to stay longer in the feeder also carry genetic effects that make their mates to shorten their occupation time. The occupation time is a trait with an upper limit (24 h); thus if one animal stays longer in the feeder, its mates consequently have to stay for less time; so, as a result of its definition, a phenotypic correlation would be expected between direct and indirect phenotypic effects. Thus, the estimated genetic correlation could be a consequence of this expected phenotypic relationship.

### Hypothesised social hierarchical structure based on correlations between feeding behaviour traits

Indirect genetic effects for a given trait are relevant because of the presence of social interactions between the animals sharing the pen (Bijma, 2014), and these interactions are a direct reflection of the social structure and hierarchy within the group (Meese and Ewbank, 1973). In our study, we have not directly recorded this social structure; however, by inspecting the phenotypic correlations between traits (Table 3, below diagonal), particularly FBT, it could be possible to hypothesise a certain social structure. Therefore, based on the phenotypic correlations between FBT, it can be concluded that animals could be clustered into two groups: (1) *Dominants*: animals that eat sedately (lower feeding rate), occupying the feeder for a long time (higher occupation time), with a large number of visits (higher feeding frequency) and with shorter intervals between them (lower time between visits). (2) *Subordinates*: animals that eat more quickly, spending less time in the feeder trough, having a reduced number of visits per day and with longer intervals between visits. This clustering of animals was confirmed using a k-means algorithm on raw phenotypic FBT records; when the clustering was set to just two groups, the following means were defined for each group: Cluster 1 (556 animals) feeding rate = −0.56, occupation time = 0.60, feeding frequency = 0.53 and time between visits = −0.56; and Cluster 2 (588 animals) feeding rate = 0.53, occupation time = −0.56, feeding frequency = −0.50 and time between visits = 0.53, resembling the distribution hypothesised by analysing the phenotypic correlations.

A number of previous studies have researched the relationships between FBT and actual social hierarchy rank. This has been assessed experimentally by exploring the antagonistic interactions between growing pigs. In these studies the relationships are not exactly the same as those we have just hypothesised. Hoy *et al*. (2012) using crossbreds from Pietrain, Landrace and Duroc breeds showed that dominant animals tend to spend more time at the feeder, but in contrast with our hypothesised social hierarchy those animals had lower feeding frequency. Vargas *et al*. (1987) in another crossbred population did not find any association between the social rank and feeding frequency; however, they observed that animals with high feeding frequency tend to be those that suffer from a larger number of displacements and aggressions around the trough. Similarly, Nielsen *et al*. (1995 and 1996) in a Large White × Landrace cross did not find any association between social rank and FBT; nevertheless, in these studies it is stated that this lack of association could be a consequence of the management in their experiments. One example of these management practices could be straw provision. So, apparently, the relationships between FBT and social ranks are far from stable and constant across populations and experiments. A large number of factors have been found to influence either FBT, social ranks or the relationship between them; for example, Baumung *et al*. (2006) show that differences between breeds have a large effect on FBT. The degree of kinship in a group could also influence the antagonist interactions within that group – which in turn defines the social rank indexes – (Fels *et al*., 2012), as well as the size of the groups (Nielsen *et al*., 1996). In addition, variation in certain internal physiological mechanisms, for example, satiety, might play an important role in the relationship between FBT and social rank position (Maselyne *et al*., 2015). This variety of results is one of the arguments used by Boumans *et al*. (2018) to propose simulation models as a tool to study the relationships between performances, social rank and FBT.

At a genetic level, given the higher estimation errors, not all the characteristics of the subordinates and dominant animals can be consistently defined. Thus, we could only postulate that dominant animals might carry genetic factors which cause them to eat at a slower pace for longer, and that they carry genetic factors which lead to a larger number of visits to the trough and to a reduced feeding interval. The consideration of AM-IGE will allow further assessment of whether the proposed hierarchy of the population is compatible with the estimated genetic correlations between IGEs within and across FBT. Dominant animals with genes which induce them to stay longer at feeder will also carry genes forcing their mates to play a subordinate role. These subordinates have them a shorter occupation time (negative correlation between DGE and IGE in Table 2), a shorter feeding rate (negative correlation between IGE of occupation time and IGE of feeding rate in Table 4) and lower time between visits (negative correlation between IGE of occupation time and IGE of time between visits in Table 4). Dominant animals will have DGE for increased feeding frequency, and they will induce a genetic effect on their subordinate mates, which supposes a reduction of this trait (negative correlation between IGE and DGE in Table 2). Based on the postulated phenotypic structure we have previously indicated, time between visits of subordinate animals should be increased in parallel to the decline of feeding frequency; this is confirmed by the strong negative (−0.99) correlation between IGE of time between visits and IGE of feeding frequency (Table 4). Dominant animals should have DGEs that reduce their own feeding rate and increase the feeding rate of their mates, and it would be expected that these subordinate animals would have an increased time between visits. Thus, a positive genetic correlation between IGE of feeding rate and IGE of time between visits would be expected, but a negative value has been estimated (−0.51). The available information does not allow us to further explain why this mismatch to our proposed structure happened. However, one simple explanation could be that some of the aforementioned correlations could have been over- or underestimated, and they do not allow for a fully coherent interpretation of the results. Another point, already mentioned, could be that other factors, beyond the social structure, influence the genetic and phenotypic correlations between traits. In spite of these exceptions we believe the overall scenario still applies and is therefore relevant: some animals capitalise the feeder, and others have to adapt to this situation.

Based on the previous discussion, it can be concluded that the parameter estimates of the multivariate models considering IGE are in some cases, but not always, compatible with a hypothesised social hierarchy structure assuming dominant and subordinate animals within pens. A better validation of the estimated model parameters would have been achieved if the actual social hierarchy had been properly assessed. Based, for example, on experiments measuring the degree of antagonistic interactions between pairs of pen mates, in order to define for each animal a social rank index value (one example of such indexes was proposed by Lee *et al*. (1982)) that could be treated as another trait can be explored for its correlations with PT and FBT.

### Is the hypothesised social hierarchical structure evidenced in the relationships between feeding behaviour and production traits?

One of most relevant points in this study was to assess the role that FBT (as proxies of a hypothesised social hierarchy) might play in the genetic evaluation for PT. In this regard, the first assessment we will conduct will be to explore whether the hypothetical social structure defined by the phenotypic correlation among FBT is the major factor responsible for the correlation structure among PT. It can be anticipated that the structure among FBT is not transferred to PT. This can clearly be seen in the phenotypic correlations between feeding rate and all PT, which have the same sign (always positive) as the correlations between occupation time and the PT, when according to the hypothesised social hierarchy structure, correlations of opposite sign would be expected (the phenotypic correlation between feeding rate and occupation time is negative).

One could also expect dominant animals (with low feeding rate according to our hypothesis) to be those having the larger ADC (Hoy *et al*., 2012), but in light of observed phenotypic correlations this is not the case. These discrepancies, as those previously discussed among FBT, imply that in the definition of the correlations between FBT and PT, other factors beyond the hypothesised social hierarchy have intervened. For those genetic correlations estimated with the AM between FBT and PT being statistically different from zero, the same discrepancy between the postulated social structure and PT was found. Thus, we can only claim, as before, that these positive correlations between ADC, ADG and BF with feeding rate must be connected to some physiological mechanisms, and not only to the fact that the genes involved in defining the position within the social rank are also responsible for greater or lesser growth, intake or backfat depth. Among the processes explaining these positive correlations with feeding rate, we could propose the physical/hormonal satiation mechanism, described in the revision by Maselyne *et al*. (2015) or further internal mechanism involving other hormonal regulations and nutritional transport processes, previously described as being involved in the feed efficiency of the animals (Reyer *et al*., 2018).

An overall assessment of the correlations between IGE and DGE across both groups of traits (FBT and PT) seems to show that the predominant direction of this correlation is from IGE of FBT to DGE of PT (Table 5). Only for this group of correlations, values of relevant magnitude were estimated. This implies that IGE of FBT could determine PT, but IGEs of PT do not seem to influence FBT. The interpretation of the observed correlations in light of the postulated social hierarchical structure is extremely complex, and will not be made, given that the proposed social structure is just a hypothesis that has not always shown coherence with the estimated correlations. Nevertheless, the overall relationships between IGE and DGE among PT and FBT can be used to interpret finds in previous studies. Ragab *et al*. (2018) used FBT as predictors in models fitting IGE of PT, in particular ADG, aiming to improve the prediction performances of the AM-IGE. The limited gain in prediction quality observed in their study (4 % to 5 %) can be explained because only IGE of FBT seems to be related to DGE of PT. No relevant correlations have been declared the other way around, that is, between IGE of PT and DGE of FBT, which would be the relevant parameter to improve accuracy in the model proposed by Ragab *et al*. (2018).

Until now, our discussion has been driven by a hypothetical social structure, predicted from the phenotypic correlations, and this structure has been used to explain the obtained genetic correlations between traits, and their genetic components of traits (IGE and DGE). We have already indicated that not having the actual social structure in our population is an important limitation for interpreting our results. The other major limitation of our study is associated with the fact that a small dataset has been used to estimate correlation parameters in complex models. Thus, in some cases, large estimation errors have been reported. Nevertheless, when the estimates are precise enough to allow for properly defining the sign of the correlations, in many cases the reported estimates in relation to FBT are compatible with the proposed hypothesis.

### Animal model fitting indirect genetic effects enriches the relationship between traits offering further selection index possibilities

Beyond the role that FBT might have played in the definition of PT correlation structure and how this relationship is established, another important result that deserves discussion is the fact that the consideration of multivariate models fitting IGEs would enrich the relationships between traits. This enriched structure could allow for the definition of selection indexes that might better address a given breeding goal. For example, with regard to PT, it was observed from additive genetic correlations obtained using the AM that it could be difficult to envisage selection indexes to indirectly improve FCR by reducing BF while increasing ADG (see genetic correlations in Table 3). However, by considering AM-IGE and differentially weighting IGE and DGE predictions, it could be possible to identify combinations between DGE and IGE (other than the often-used total breeding value prediction (Bijma, 2014)) that might yield a stronger direct response on ADG and BF and an indirect response on FCR. One example could be to select for reducing BF through its IGE, positively correlated with FCR IGE (0.75), while ADG could be improved by selection solely on its DGE, which has a lower correlation with DGE of FCR (0.13). In this way we would implicitly be considering a selection index accounting both for PT and welfare and FBT (Ellen *et al*., 2014).

As an overall result we can indicate that the extension of the bivariate model relating PT and FBT to AM-IGE has evidenced that the genetic correlations between these two groups of traits, as well as within type of trait, are more complex than would be expected from the analysis with the AM. To our knowledge, this type of bivariate analysis has not been performed so far, so we cannot contextualise our particular results. However, based on our results we could examine possible benefits of additional selection indexed combining IGE and DGE considering alternatives weighting across traits and effects. These alternative indexes might yield stronger responses in the selection objective than would be expected from indexes based on animal model breeding value predictions or total breeding value predictions from the AM-IGE.

## Conclusions

For the two types of traits considered in this study (PT and FBT), models fitting IGEs were statistically preferable over the traditional animal model. The consideration of such models improves the genetic correlation structure between the involved traits; thus opening up the possibility of proposing indexes drives genetic responses for breeding goals which cannot be defined under the traditional animal model. Our results also show that the relationship between FBT can partially be attributed to a social hierarchical structure, but this structure only explains a certain proportion of the genetic relationships between PT and between FBT and PT, particularly those involving IGE of FBT and DGE of PT.

## Software and data repository resources

The datasets and programmes used in the current study are available from the corresponding author on reasonable request.
